# Influence of Rifamycin on Survival in Patients with Concomitant Lung Cancer and Pulmonary Tuberculosis

**DOI:** 10.3390/biomedicines11123130

**Published:** 2023-11-24

**Authors:** Ho-Sheng Lee, Yu-Feng Wei, Chin-Chung Shu

**Affiliations:** 1 Department of Internal Medicine, E-Da Hospital, I-Shou University, Kaohsiung 82445, Taiwan; leehoshn@gmail.com; 2 School of Medicine for International Students, College of Medicine, I-Shou University, Kaohsiung 82445, Taiwan; 3 Department of Internal Medicine, E-Da Cancer Hospital, I-Shou University, Kaohsiung 824005, Taiwan; 4 Department of Internal Medicine, National Taiwan University Hospital, No. 7, Chung Shan South Road, Taipei 100225, Taiwan

**Keywords:** rifamycin, tuberculosis, lung cancer, survival

## Abstract

Background: The coexistence of lung cancer and tuberculosis is not rare. Rifamycin plays a pivotal role in anti-tuberculosis therapy. However, its potential impact on the liver metabolism of oncology drugs raises concerns. We performed this study to explore whether Rifamycin affects the survival of patients with tuberculosis and lung cancer. Methods: Drawing from the Taiwan National Health Insurance Research Database, we identified patients diagnosed with concurrent lung cancer and tuberculosis between 2000 and 2014. Patients were categorized based on whether they underwent rifamycin-inclusive or rifamycin-exempt anti-tuberculosis therapy. Subsequently, we paired them at a 1:1 ratio and evaluated the mortality risk over a two-year span. Results: Out of the study participants, 1558 (81.4%) received rifamycin-based anti-tuberculosis therapy, while 356 (18.6%) underwent a rifamycin-free regimen. Analysis revealed no marked variance in the biennial mortality rate between the groups (adjusted hazard ratio: 1.33, 95% confidence interval 0.93–1.90, *p* = 0.1238). When focusing on the matched sets comprising 127 individuals in each group, the data did not indicate a significant link between rifamycin and a heightened two-year mortality risk (adjusted hazard ratio: 1.00, 95% confidence interval 0.86–1.18, *p* = 0.9538). Conclusions: For individuals with concomitant lung cancer and tuberculosis, rifamycin’s administration did not adversely influence two-year survival. Thus, rifamycin-containing anti-TB regimens should be prescribed for the indicated patients.

## 1. Introduction

Lung cancer (LC) is the leading cause of cancer death globally [1]. According to the Taiwan annual cancer report from 2019, in that year, LC was the second most common cancer and the leading cause of cancer death. Also in that year, with 16,233 new cases being reported, the annual incidence stood at 68.77 per 100,000 individuals [2]. LC occurs in middle-aged and older individuals. The median age of LC diagnosis was reportedly 66 years old in Taiwan in 2019. Patients usually have minimal symptoms with early-stage LC. Mild symptoms such as cough, shortness of breath, or chest discomfort are frequently ignored by patients. Older people commonly regard these mild airway symptoms as normal conditions in normal aging, and they usually search for medical advice when hemoptysis or more severe disease symptoms, e.g., those that cause significant impairments in exercise ability and daily living, are noticed. By the time of diagnosis, most patients with LC are already at an advanced stage and incurable. In early-stage LC, patients have the chance to be cured via surgery or radiotherapy. For patients with incurable advanced lung cancer, palliative chemotherapy or targeted therapy are the mainstays of treatment. If the patient is too ill for these therapies, hospice care is another treatment option.

Tuberculosis (TB), caused by *Mycobacterium tuberculosis* infection, is one of the most common infectious diseases and leading causes of mortality worldwide [3]. The prevalence of TB differs by region. TB infection is more common in developing countries. Taiwan is also an area wherein TB infection is endemic. There were 7062 new cases (30.1 cases in every 100,000 persons) of TB and 442 deaths associated with TB in Taiwan in 2021, according to the epidemiology data of tuberculosis reported by the Taiwan CDC [4]. In Taiwan, most TB infections involve the reactivation of TB. Most patients have asymptomatic latent TB infection in young age, and TB reactivates when patients become old or less immunocompetent. Notably, TB is a risk factor for LC, with a hazard ratio (HR) of 1.37 to 1.49 [5]. The incidence of pulmonary tuberculosis is about 2–5% in lung cancer patients [6]. This could be caused by the TB-related chronic inflammation and the common characteristics shared by LC and TB patients, such as old age and the tendency to smoke cigarettes.

Posing a hazardous risk to public health due to their disease’s infectious nature, TB patients are treated according to strict guidelines. Patients usually take a combined regimen for initial treatment, consisting of rifampicin, ethambutol, isoniazid, and pyrazinamide. This treatment regimen can be adjusted further according to the patient’s treatment response and side effects. Among the anti-TB medications, rifamycin, including rifampicin and rifabutin, is an important first-line drug in eradicating *Mycobacterium tuberculosis* [7]. Rifamycin has potent bactericidal activity against *Mycobacterium tuberculosis* in both pulmonary TB and extrapulmonary TB. It inhibits the Deoxyribonucleic acid (DNA)-dependent Ribonucleic acid (RNA) polymerase enzyme, which is crucial for RNA synthesis. Through using this drug in combination with other medications, we can shorten treatment courses and prevent drug resistance.

However, rifamycins are strong inducers of cytochrome P-450 (CYP) enzymes [8], which subsequently enhance the metabolism of some pivotal agents for LC treatment, e.g., epidermal growth factor receptor–tyrosine kinase inhibitors (EGFR-TKIs) and other TKIs [9,10,11], and chemotherapy agents such as docetaxel, paclitaxel, and vinorelbine [12,13]. In addition, although therapeutic monoclonal antibodies of immunotherapy do not directly interact with CYP enzymes, the immune response may affect the level of CYP enzymes [14]. Therefore, the decreasing effect of anti-cancer agents with concomitant rifamycin use has raised concerns about survival among patients with LC and TB worsening due to rifamycin use. Few studies have investigated this issue. Hence, we conducted the present study to analyze the influence of rifamycin use on two-year survival among patients with concomitant LC and pulmonary TB.

## 2. Materials and Methods

### 2.1. Data Source

We searched for data in the National Health Insurance Research Dataset (NHIRD) from 2000–2016. The NHIRD is a dataset of insurance claims provided and maintained by the Collaboration Center of Health Information Application of the Ministry of Health and Welfare of Taiwan. The NHIRD is a national database that includes all medical claims by NHI in Taiwan, including hospitalization and outpatient services for all of the nation’s 23 million people. It is one of the largest databases of medical information in the world.

The collected data encompass demographic details, clinical visitation dates, diagnosis codes, prescription details, and related expenditures. Notably, information on individuals’ body mass index and lifestyle habits was not incorporated until 2011. All diagnoses are classified according to the International Classification of Diseases, 9th Revision, Clinical Modification (ICD-9-CM) or the International Statistical Classification of Diseases and Related Health Problems, 10th Revision (ICD-10). Each outpatient visit claim contains three diagnosis fields, whereas inpatient claims have five. Access to the NHIRD is restricted, necessitating formal requests by researchers. Due to NHIRD regulations, the data generated and analyzed in this study are not publicly available.

### 2.2. Ethics Statement

Our research protocol secured approval from the E-Da Hospital’s Institutional Review Board under the reference EMRP-108061. The data had already been stripped of personal identifiers before it was provided to researchers from the NHIRD, ensuring anonymity. There was no requirement for the provision of informed consent for this investigation, according to the Institutional Review Board. All procedures in the study adhered strictly to pertinent regulations and the Declaration of Helsinki.

### 2.3. Identification of Lung Cancer Cases

Taiwan’s National Health Insurance (NHI) program aids cancer patients by defraying potential medical expenses post-diagnosis. Given the significant expenses related to cancer treatments, stringent criteria are set. Hospitals are mandated to present compelling proof, such as pathology findings and diagnostic imagery, in order to validate a cancer diagnosis to ensure that the patient is reimbursed. A detailed compilation of data about cancer patients is maintained in the Registry of Cancer Patient Database. Leveraging the rigorous validation process of the database, we extracted data to pinpoint individuals diagnosed with LC. (ICD-9 codes: 162.2, 162.3, 162.4, 162.5, 162.8, 162.9; ICD-10 codes: C34.0, C34.1, C34.2, C34.3, C34.8, and C34.9).

### 2.4. Identification of Active Tuberculosis Cases

In Taiwan, every individual diagnosed with active TB is registered within the National Infectious Disease Reporting System. Diagnosis requires a combination of clinical, radiological, and either microbiological or pathological evidence of TB infection. Following a confirmed TB diagnosis (ICD-9 codes: 011.90 and 011.91; ICD-10 codes: A15.0–A15.9), treatment is aligned with recognized guidelines [15], with public health nurses supervising the administration of anti-TB medication to ensure patient adherence.

### 2.5. Patient Selection, Exclusion, and Grouping

From the insurance claims records, we identified all patients diagnosed primarily with LC between 2000 and 2014 and acquired all relevant data through to 2017. Each selected patient was followed for a span of two years or until their death. Those excluded included individuals below the age of 20, those diagnosed with other cancers or specific catastrophic illnesses listed by the NHI, those with an unclear diagnosis date, those with rifamycin prescriptions unrelated to TB, those who underwent rifamycin treatment for less than 3 months, deaths concurrent with LC diagnosis, or those with an absence of TB diagnosis spanning from three months before to three months post LC diagnosis. Patients with liver injury were excluded because rifamycin is notorious for being associated with liver toxicity, which might influence survival. The selected patients were bifurcated into two cohorts: those treated with rifamycin-based regimens (LCTB-R) and those who underwent therapy devoid of rifamycin (LCTB-Rf).

### 2.6. Matched Comparison

Acknowledging the inherent variability in initial clinical attributes, we executed a 1:1 matched analysis anchored on criteria such as age, gender, index day, LC stage, and associated health conditions (via propensity scores). The subsequent computations adhered to the methods outlined in the following section.

### 2.7. Data Analysis

We initiated the analysis by examining baseline traits and accompanying health conditions. Drug usage rates were determined for the entire observation duration. Prescription records detailed order dates, dosages, administration methods, and the duration of each medication. The reference index day was assigned on the LC diagnosis date. Comorbidities were labeled based on their presence before the index day. The following period was finished by a patient’s exit from the NHI program, death, or at least two years of follow-up. To discern mortality risks, we deployed a segmented Cox regression hazard model, calibrating for demographics such as age, gender, LC stage, and concurrent health conditions. All computational procedures and analytics utilized the Statistical Analysis System software for Windows (version 9.4, SAS Institute, Cary, NC, USA).

## 3. Results

### 3.1. Patient Recruitment

**Figure 1:**
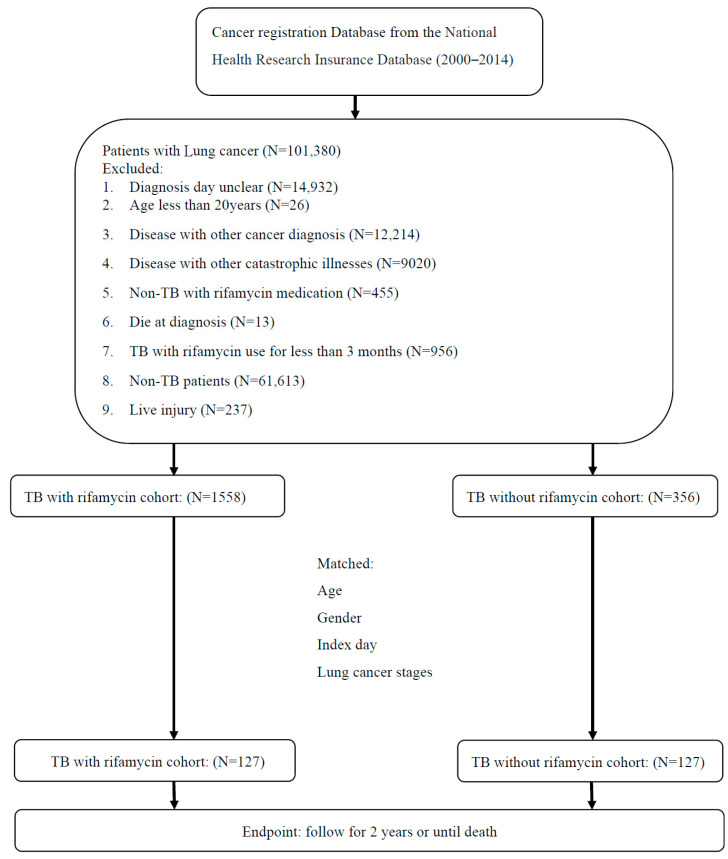
Study flow diagram of patient recruitment. Abbreviations: TB: tuberculosis; LCTB-R: lung cancer and tuberculosis with rifamycin-containing treatment; LCTB-Rf: lung cancer and tuberculosis with rifamycin-free treatment.

A total of 101,380 patients with LC were diagnosed from 2000 to 2014, of whom 99,229 were excluded due to the following reasons: unclear date of diagnosis (N = 14,932), age younger than 20 years (N = 26), disease concomitant with other cancers (N = 12,214), disease concomitant with other catastrophic illnesses (N = 9020), non-TB patients (N = 61,613), rifamycin use other than for TB (N = 455), rifamycin use for less than 3 months (N = 956), death at the diagnosis of LC (N = 13), and liver injury (N = 237) (Figure 1). Finally, 1558 patients were included in the LCTB-R group, and 356 patients comprised the LCTB-Rf group. After matching to facilitate a matched comparison analysis, there were 127 patients in each group.

### 3.2. Comparisons of Variables between the LCTB-Rf and LCTB-R Groups

The baseline characteristics of the enrolled patients are shown in Supplementary Table S1. The LCTB-Rf group was older than the LCTB-R group (mean± standard deviation, 68.65 ± 13.14 vs. 66.94 ± 13.38 years, *p* = 0.0293). In addition, the LCTB-Rf group had a higher prevalence of stage III LC and higher BMI values than the LCTB-R group (stage I & II, 13.21% vs. 14.7%; stage III, 24.72% vs. 20.35%; stage IV, 55.90% vs. 55.91%, *p* = 0.0118; BMI, 23.18 ± 3.84 vs. 22.25 ± 3.48 kg/m^2^, *p* = 0.0117). There were no significant differences between the LCTB-Rf and LCTB-R groups in sex (male 69.66% vs. 69.58%), cigarette smoking (83.99% vs. 86.39%, *p* = 0.2390), and histology (adenocarcinoma 49.44% vs. 52.18%; squamous cell carcinoma 27.81% vs. 22.14; *p* = 0.0856).

With regard to medications, higher prescription rates of CYP inhibitors (21.91% vs. 14.96%, *p* = 0.0013) and P-glycoprotein (P-gp) inhibitors (15.73% vs. 9.31%, *p* = 0.0004) were noted in the LCTB-Rf group. In terms of comorbidities, the LCTB-Rf group had a higher prevalence of congestive heart failure (11.24% vs. 7.57%, *p* = 0.0235), chronic lung disease (70.51% vs. 56.68%, *p* < 0.0001), gastrointestinal ulcer disease (48.03% vs. 41.66%, *p* = 0.0285), diabetes mellitus (33.71% vs. 22.72%, *p* < 0.0001), and diabetes mellitus with end organ damage (11.52% vs. 6.42%, *p* = 0.0009).

### 3.3. Multivariable Analysis for Two-Year Survival

Two-year mortality was similar between the LCTB-R and LCTB-Rf groups, as shown in Supplementary Table S2 (adjusted hazard ratio [aHR] 1.33; 95% confidence interval [CI] 0.93–1.90; *p* = 0.1238, LCTB-Rf group as reference). Stratified Cox proportional regression hazards analysis demonstrated that CYP inducers (aHR 2.73, 95% CI 1.54–4.85, *p* = 0.0006), EGFR TKIs (aHR 1.79, 95% CI 1.18–2.73, *p* = 0.0065), advanced-stage LC, and moderate or severe kidney disease (aHR 2.17, 95% CI 1.07–4.40, *p* = 0.0328) were associated with a higher risk of two-year mortality.

### 3.4. Validation Using Matched Cohorts

To correct for the heterogeneity in the baseline clinical characteristics, we conducted a matched comparison analysis at a ratio of 1:1 (due to the small number of cases). After matching for age, sex, index date, LC stage, and comorbidities (propensity score), 127 pairs of patients were included for analysis.

Comparisons of the matched LCTB-Rf and LCTB-R groups are shown in Table 1. Mean age (71.86 ± 9.61 vs. 71.5 ± 9.66 years), sex distribution (males 77.17% in both groups), lifestyle habits (smoking [84.25% vs. 88.98%], alcohol consumption [66.93% vs. 74.80%]), and LC stage (stages 1 & 2: 7.09%, stage 3: 19.69%, stage 4: 70.08% in both groups) were similar.

The distribution of histology type was similar (adenocarcinoma 51.97% vs. 52.76%, squamous cell carcinoma 32.28% vs. 27.56%). The use of medications including CYP inducers, CYP inhibitors, P-gp inducers, P-gp inhibitors, proton pump inhibitors (PPIs), and EGFR TKIs was also similar. In comorbidities, the LCTB-Rf group had higher rates of congestive heart failure (16.54% vs. 6.30%, *p* = 0.0103), chronic lung disease (73.23% vs. 59.06%, *p* = 0.0170), diabetes mellitus (31.50% vs. 18.11%, *p* = 0.0135), and diabetes with end organ damage (11.02% vs. 3.94%, *p* = 0.0318) than the LCTB-R group.

**Figure 2:**
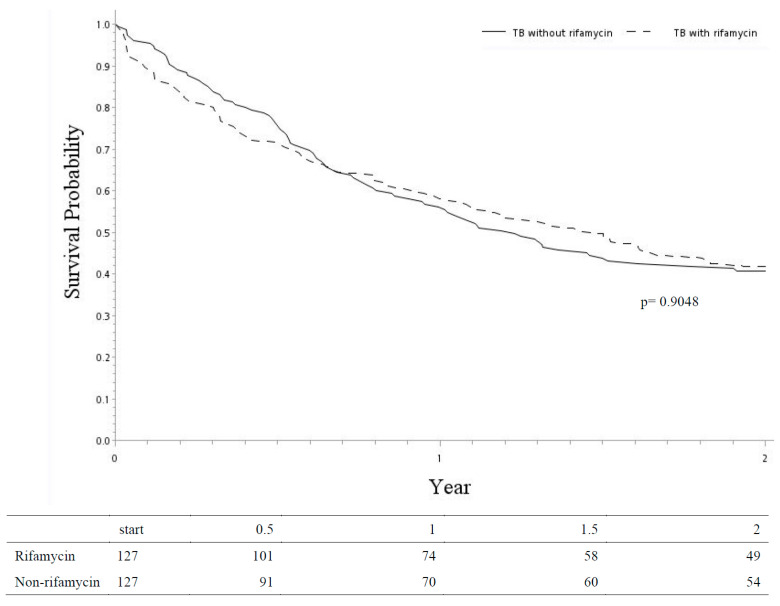
Kaplan–Meier curves for two-year survival in LCTB-R and LCTB-Rf matched comparisons. Abbreviations: LCTB-R: lung cancer and tuberculosis with rifamycin-containing treatment; LCTB-Rf: lung cancer and tuberculosis with rifamycin-free treatment.

In survival analysis, the Kaplan–Meier curve method showed two overlapping curves (Figure 2), and the median overall survivals were about 1.5 years in both groups. The two-year mortality rate was similar between the two groups (aHR 1.00, 95% CI 0.86–1.18, *p* = 0.9538) (Table 2). Regarding the predictors of mortality, male gender (aHR 1.46, 95% CI 1.27–1.69, *p* < 0.0001), CYP inducers (aHR 1.46, 95% CI 1.17–1.84, *p* = 0.0010), CYP inhibitors (aHR 1.35, 95% CI 1.15–1.58, *p* = 0.0002), P-gp inhibitors (aHR 1.29, 95% CI 1.06–1.58, *p* = 0.0101), PPIs (aHR 1.31, 95% CI 1.15–1.50, *p* = 0.0001), and EGFR TKIs (aHR 1.93, 95% CI 1.66–2.25, *p* < 0.0001) were associated with an increased risk of mortality. 

Lower mortality rates were observed in the patients with cerebrovascular disease (aHR 0.83, 95% CI 0.69–0.99, *p* = 0.0397). Patients with stage 3 or stage 4 LC have a higher risk of death compared to those with stage 1 & 2 LC.

## 4. Discussions

In this national database study, we investigated the two-year survival of patients with concomitant LC and TB at initial cancer diagnosis. In patients with LC and TB, their survival was determined by LC in most of the cases due to the poorer prognosis of LC. To thoroughly evaluate the influence of rifamycin with minimal impact of LC, we need to be sure that the diagnoses of LC and TB were made in the same time frame and that the patients had enough exposure to rifamycin. We made strict exclusion criteria. We excluded patients with a time span of more than 3 months between their diagnosis of LC and TB, and patients who had used rifamycin for less than 3 months. Although there were 101,380 patients diagnosed with LC between 2000 and 2014, after filtering with the exclusion criteria, there were only 1558 (81.4%) patients in the rifamycin treatment group (LCTB-R group) and 356 (18.6%) patients in the rifamycin-free group (LCTB-Rf group). Compared with other cancer patients, LC patients are older, with an average age around 66 years old at diagnosis, and have worse survival rates, especially among patients with advanced-stage LC. These confounding factors had a great impact on survival, especially in the cohort with a small number of cases and heterogeneous characteristics. To eliminate such differences, we conducted the matched comparison analysis.

We matched age, gender, cancer stage, and index day in 1:1 ratio between the two groups. Index day refers to the date that the patient’s LC diagnosis was made. The treatment of LC has hugely improved in recent years, especially after TKI was introduced in Taiwan. Gefitinib was launched in Taiwan in 2003, and erlotinib was launched in 2006. Two-year survival increased by 19.81% in the 3 years following the launch of gefitinib [16]. In another study, 3-year survival improved from 26.57% to 36.48% between 2010 and 2014 [17]. Matching the index day could exclude the difference related to the evolution of LC treatment in different years and its related influence on patient survival. There were only 127 patients enrolled in each arm in the matched comparison analysis, but this is already one of the largest cohorts for a study, as far as we know.

Before our matched comparison analysis, there were some significant differences in the baseline clinical characteristics between the two cohorts, including in age, LC stage, and prescription of CYP inhibitors and P-gp inhibitors, as shown in Supplementary Table S1. The patients in the LCTB-Rf group had a higher risk of comorbidities, including congestive heart failure, chronic lung disease, gastrointestinal ulcers, and diabetes mellitus (DM). There were also static differences in the distribution of lung cancer stage and BMI between the two groups, but the differences were too small to have a clinical impact. Generally speaking, the LCTB-Rf cohort were older and weaker, being prescribed more medications.

After investigating two-year survival in the matched comparison analysis, we found that the use of rifamycin had no statistically significant difference on survival. Rifamycin-containing multi-drug regimens for at least 6 months are recommended in the current treatment guidelines for TB [15,18]. Rifamycin is known to induce several side effects, including orange-colored urine, liver toxicity, gastrointestinal upset, nausea, and possible allergic reactions. Clinicians should be cautious when prescribing rifamycin to patients with impaired liver function or underlying liver disease, alcohol dependence, multiple comorbidities, and long-term medications, as well as pregnant patients and those that are breast feeding. The side effects and adherence to anti-TB medications are major concerns in patients with concomitant TB and LC because of the drug–drug interactions between rifamycin and anti-cancer medications. In the present study, the patients in the LCTB-Rf group had more comorbidities, including congestive heart failure, chronic lung disease, and DM. If a patient has multiple comorbidities, it might discourage clinicians to utilize rifamycin on the patients. Clinicians have also managed to decrease the negative influence of rifamycin [19] by adjusting anti-cancer drugs to minimize drug–drug interaction or maximize the time gap between dosing rifamycin and anti-cancer medication.

The benefits of rifamycin in TB treatment and the prevention of TB recurrence might be masked by the short survival of LC. Most of the enrolled patients had advanced LC (in the matched analysis, stage IV, 73.55%; stage III, 20%). The median survival was 1.5 years among the patients in this study, non-inferior to the overall national survival of advanced LC, which was reportedly a median survival of 10.7 months in stage IV LC patients and 14.8 months in those with stage IIIB LC between 2010–2016 in a Taiwan lung cancer cohort study [16].

In the matched cohort analysis, male gender was associated with an increased risk of death, compatible with the national database study: the 5-year survival of LC was better in female patients, with 13.28% in men and 20.67% in women [16]. The concomitant use of CYP inducers, CYP inhibitors, and P-gp inhibitors was associated with a higher mortality rate. Rifamycins are strong inducers of CYP enzymes and the P-gp efflux pump [20], resulting in increasing clearance and decreasing the bioavailability of the medications metabolized by CYP-450 enzymes, such as beta blockers, nonsteroidal anti-inflammatory drugs, omeprazole, statins, zolpidem, and TKIs [21]. It is confusing that both inducers and inhibitors of CYP were associated with mortality despite their opposing mechanisms. This may reflect the multiple comorbidities of patients rather than any pharmacological effects. In addition, although this is a national database study, after matching, there were only 127 patients in each arm, and only 10–27 patients were in the subgroup consisting of those that have used CYP inducers/inhibitors and P-gp inducers/inhibitors. With such a small number of cases, one or two outlying patients might have had a decisive influence on the results of our analysis.

PPIs were associated with an increased risk of death in this study. PPIs can sustain the gastric environment pH < 6.0, reduce the rebleeding of gastric ulcers, and prevent stress ulcers. PPIs were widely used for their adequate effects and safety. However, the long-term use of PPIs may interfere with nutrient absorption and drug action and change the microbiota of the human body, thereby reducing the anti-cancer effect of LC treatment and influencing treatment outcomes. There is some controversy surrounding the influence of PPIs on survival among LC patients receiving active systemic anti-cancer treatment. In a meta-analysis, PPIs were associated with higher mortality and shorter PFS and OS in patients with aggressive systemic anti-LC treatment, including targeted therapy, chemotherapy, and immunotherapy [22]. In the post hoc analysis of the OAK and POLAR trials, LC patients adminstered a combination of chemotherapy and atezolizumb had worse outcomes with PPI use [23]. Sustaining a low pH may weaken our defense to some microorganisms, and an increased risk of TB infection has been observed in clinical studies [24,25]. These findings are compatible with those of our study.

EGFR TKIs were associated with an increased hazard ratio of death in our study. EGFR TKIs are used widely in advanced LC with EGFR gene mutation, with better disease control rates and progression-free survival rates than standard chemotherapy. The survival of LC patients was much improved after EGFR TKIs were introduced in [16]. In other words, EGFR-TKI is a surrogate marker of stage IIIB or stage IV LC; therefore, it was associated with decreased survival in the present study.

Regarding comorbidities, in our study, chronic lung disease, gastrointestinal ulcers, DM, cerebrovascular disease, and congestive heart failure were the most common, in alignment with other large national database studies [26,27]. Upon further analysis, most of the comorbidities did not have significant association with the risk of death, except for cerebrovascular disease, which was associated with a decreased risk of death. This is different to other studies showing a relationship between comorbidities and poor survival among LC patients, and cerebrovascular disease has been reported to be one of the most significant death-associated comorbidities in several large database studies [26,27].

To avoid rifamycin-related liver injury influencing patient survival, we excluded patients with acute liver damage (237 patients in total). We performed an analysis of these 237 patients, and their clinical characteristics are shown in Supplementary Table S3, while an analysis of their risk factors for death are shown in Supplementary Table S4. In these patients, 62 (26.16%) were rifamycin-free, and 175 (73.84%) received rifamycin treatment. Considering the liver injury patients and the study-enrolled cohort together, the incidence of liver injury was not higher in the rifamycin-receiving population (62/[62 + 356] = 14.83% in the non-rifamycin population, 175/[175 + 1558] = 10.10% in the rifamycin-receiving population). In the Cox regression hazard model of occurrence of death in the liver injury population, rifamycin use had no statistically significant influence on risk of death (Supplementary Table S4).

There are several limitations to this study. First, as this was a retrospective national database study, there was no standardized protocol for patient treatment. In addition, although this is the largest cohort study of patients with concomitant LC and TB and matched comparison analysis was conducted to exclude uneven confounding factors, the number of cases was still too small to give a solid conclusion, and further large and prospective studies are needed to validate the impact of rifamycin use on LC patients. Third, types of EGFR mutations and other driver mutations were not available in the database. Fourth, targeted therapies other than EGFR TKIs and immunotherapy agents had not been approved in Taiwan during the time frame of this study.

In the future, prospective studies are needed to confirm the influence of rifamycin in patients with concomitant LC and TB, to find out the susceptible patient subgroups, and to improve standard treatment protocols. The development of better anti-TB and anti-LC medications may reduce side effects and drug–drug interactions and improve patient survival. However, although the annual incidence of LC is increasing in Taiwan, the prevalence of TB is decreasing. The annual incidence of TB dropped from 72.5 per 100,000 populations in 2005 to 30.1 per 100,000 populations in 2021 in Taiwan [4]. It will be more difficult to enroll such large populations of patients with concomitant TB infection and LC in the future.

In conclusion, rifamycin, one important first-line anti-TB drug, is only prescribed in around 80% in patients with concomitant TB and LC. There was no significant difference in two-year mortality between the two patient groups with or without using rifamycin for TB treatment. Therefore, considering rifamycin’s great effect in terms of TB treatment, rifamycin-containing anti-TB regimens could be prescribed to patients with concomitant LC and TB. There were no concerns with respect to rifamycin causing worsened survival.

## Figures and Tables

**Table 1:** Baseline Characteristics of the study population in matched comparison.

	Non-Rifamycin N = 127	Rifamycin N = 127	*p*-Value
Age	71.86 ± 9.61	71.5 ± 9.66	0.7673
Age Group			0.4838
20–59	13 (10.24)	19 (14.96)	
60–75	58 (45.67)	52 (40.94)	
>75	56 (44.09)	56 (44.09)	
Gender			>0.9999
Female	29 (22.83)	29 (22.83)	
Male	98 (77.17)	98 (77.17)	
Medications			
CYP inducer	10 (7.87)	11 (8.66)	0.8198
CYP inhibitor	27 (21.26)	22 (17.32)	0.4266
P-gp inducer	10 (7.87)	5 (3.94)	0.1832
P-gp inhibitor	22 (14.19)	16 (10.32)	0.2434
PPI	45 (35.43)	38 (29.92)	0.349
ALK TKI			
EGFR TKI	20 (15.75)	17 (13.39)	0.5936
Lung cancer stages			>0.9999
Stage 1 & 2	9 (7.09)	9 (7.09)	
Stage 3	25 (19.69)	25 (19.69)	
Stage 4	89 (70.08)	89 (70.08)	
Unknow	4 (3.15)	4 (3.15)	
Histology			0.4679
Adenocarcinoma	66 (51.97)	67 (52.76)	
Squamous cell carcinoma	41 (32.28)	35 (27.56)	
Small cell carcinoma	5 (3.94)	12 (9.45)	
Unclassified malignancy	11 (8.66)	10 (7.87)	
Other histological types	4 (3.15)	3 (2.36)	
BMI	23.19 ± 3.74	22.16 ± 3.25	0.1741
Habit			
Smoking	107 (84.25)	113 (88.98)	0.2689
Betel nut	82 (64.57)	92 (72.44)	0.1768
Alcohol	85 (66.93)	95 (74.80)	0.1673
Comorbidities			
Myocardial infarct	12 (9.45)	5 (3.94)	0.0788
Congestive heart failure	21 (16.54)	8 (6.30)	0.0103
Peripheral vascular disease	5 (3.94)	6 (4.72)	0.7579
Cerebrovascular disease	28 (22.05)	28 (22.05)	>0.9999
Dementia	6 (4.72)	3 (2.36)	0.3086
Chronic lung disease	93 (73.23)	75 (59.06)	0.0170
Connective tissue disease	4 (3.15)	4 (3.15)	>0.9999
Ulcer	63 (49.61)	66 (51.97)	0.7065
Chronic liver disease	23 (18.11)	19 (14.96)	0.4993
Diabetes	40 (31.50)	23 (18.11)	0.0135
Diabetes with end organ damage	14 (11.02)	5 (3.94)	0.0318
Moderate or severe kidney disease	12 (9.45)	4 (3.15)	0.0388
Death	73 (57.48)	78 (61.42)	0.5228

Abbreviations: BMI: body mass index; CYP: cytochrome P450; EGFR: epidermal growth factor receptor; TKI: tyrosine kinase inhibitor; P-glycoprotein; PPI: proton pump inhibitor.

**Table 2:** Prediction for occurrence of death in matched comparison analysis.

	Crude	*p*-Value	Adjusted	*p*-Value
	HRs		HRs	
Rifamycin vs. non-rifamycin	0.95 (0.81–1.10)	0.4800	1.00 (0.86–1.18)	0.9538
Age	1.00 (1.00–1.01)	0.0377	1.01 (1.00–1.01)	0.0353
Male vs. Female	1.50 (1.31–1.73)	<0.0001	1.46 (1.27–1.69)	<0.0001
Medications				
CYP inducer	1.55 (1.28–1.87)	<0.0001	1.46 (1.17–1.84)	0.0010
CYP inhibitor	1.58 (1.36–1.84)	<0.0001	1.35 (1.15–1.58)	0.0002
P-gp inducer	1.30 (1.05–1.59)	0.0143	1.04 (0.82–1.33)	0.7465
P-gp inhibitor	1.47 (1.22–1.77)	<0.0001	1.29 (1.06–1.58)	0.0101
PPI	1.37 (1.21–1.56)	<0.0001	1.31 (1.15–1.50)	0.0001
EGFR TKI	2.12 (1.83–2.45)	<0.0001	1.93 (1.66–2.25)	<0.0001
Lung cancer stages				
Stage 1 & 2	REF.		REF.	
Stage 3	2.87 (2.23–3.71)	<0.0001	2.58 (2.00–3.35)	<0.0001
Stage 4	2.85 (2.25–3.60)	<0.0001	2.48 (1.95–3.16)	<0.0001
Unknown	1.93 (1.41–2.64)	<0.0001	1.90 (1.39–2.61)	0.0001
Comorbidities				
Myocardial infarct	0.89 (0.68–1.17)	0.4046	0.96 (0.73–1.27)	0.7917
Congestive heart failure	1.11 (0.89–1.38)	0.3462	0.98 (0.77–1.24)	0.8418
Peripheral vascular disease	1.21 (0.89–1.64)	0.2259	1.18 (0.86–1.62)	0.2994
Cerebrovascular disease	0.91 (0.77–1.07)	0.2544	0.83 (0.69–0.99)	0.0397
Dementia	0.86 (0.60–1.25)	0.4387	0.96 (0.65–1.42)	0.8506
Chronic lung disease	1.07 (0.95–1.21)	0.2677	1.05 (0.92–1.20)	0.5016
Connective tissue disease	0.78 (0.47–1.29)	0.3334	0.74 (0.44–1.24)	0.2533
Ulcer	0.95 (0.84–1.08)	0.4598	0.98 (0.86–1.11)	0.7145
Chronic liver disease	0.90 (0.77–1.06)	0.2238	0.98 (0.82–1.16)	0.8068
Diabetes	0.98 (0.85–1.13)	0.8103	0.96 (0.81–1.13)	0.6336
Diabetes with end organ damage	0.92 (0.72–1.17)	0.4805	0.90 (0.69–1.19)	0.4739
Hemiplegia	1.05 (0.64–1.72)	0.8434	1.29 (0.77–2.15)	0.3362
Moderate or severe kidney disease	0.95 (0.74–1.22)	0.7012	0.91 (0.70–1.18)	0.4663
Moderate or severe liver disease	0.69 (0.19–1.87)	0.3801	0.56 (0.18–1.77)	0.3250

Abbreviations: CYP: cytochrome P450; EGFR: epidermal growth factor receptor; TKI: tyrosine kinase inhibitor; P-glycoprotein; PPI: proton pump inhibitor.

## Data Availability

All the data we have are already in the manuscript and the Supplementary Materials.

## Supplementary Materials

The following supporting information can be downloaded at: https://www.mdpi.com/article/10.3390/biomedicines11123130/s1, Table S1: Baseline characteristics of the study population (unmatched comparison); Table S2: Prediction for occurrence of death (unmatched comparison); Table S3: Baseline Characteristics of the liver injury population; Table S4: Prediction for occurrence of death in liver injury population.

## Abbreviations

CI: confidence interval; CYP: cytochrome P450; EGFR: epidermal growth factor receptor; EGFR-TKI: EGFR–tyrosine kinase inhibitor; HR: hazard ratio; ICD-10: International Statistical Classification of Diseases and Related Health Problems, 10th Revision; ICD-9-CM: International Classification of Diseases, 9th Revision, Clinical Modification; LC: lung cancer; LCTB-R: lung cancer and tuberculosis with rifamycin treatment; LCTB-Rf: lung cancer and tuberculosis with rifamycin-free treatment; NHI: National Health Insurance; NHIRD: National Health Insurance Research Database; P-gp: P-glycoprotein; PPI: proton pump inhibitor; TB: tuberculosis.
