# Supplementary material for: Influence of Rifamycin on Survival in Patients with Concomitant Lung Cancer and Pulmonary Tuberculosis
Source: Biomedicines. 2023 Nov 24;11(12):3130. doi: 10.3390/biomedicines11123130 (PMC10741138; doi:10.3390/biomedicines11123130)
Supplement: Supplementary file 1 [file biomedicines-11-03130-s001.zip › biomedicines-2613485-supplementary.pdf]

Supplementary Table S1. Baseline characteristics of the study population (unmatched comparison)

	Non-rifamycin N=356	rifamycin N=1558	p-value
Age	68.65±13.14	66.94±13.38	0.0293
Age Group			0.0788
20-59	84(23.6)	449(28.82)	
60-75	141(39.61)	614(39.41)	
>75	131(36.8)	495(31.77)	
Gender			0.9745
Female	108(30.34)	474(30.42)	
Male	248(69.66)	1084(69.58)	
Medications			
CYP inducer	32(8.99)	141(9.05)	0.9710
CYP inhibitor	78(21.91)	233(14.96)	0.0013
P-gp inducer	28(7.87)	123(7.89)	0.9851
P-gp inhibitor	56(15.73)	145(9.31)	0.0004
PPI	126(35.39)	472(30.3)	0.0611
EGFR TKI	53(14.89)	242(15.53)	0.7610
Lung cancer stages			0.0118
Stage 1 &2	47(13.21)	229(14.7)	
Stage 3	88(24.72)	317(20.35)	
Stage 4	199(55.90)	871(55.91)	
Unknow	22(6.18)	141(9.05)	
Histology			0.0856
Adenocarcinoma	176(49.44)	813(52.18)	
Squamous cell carcinoma	99(27.81)	345(22.14)	
Small cell carcinoma	18(5.06)	112(7.19)	
Unclassified malignancy	42(11.8)	166(10.65)	
Other histological types	21(5.9)	122(7.83)	
BMI	23.18±3.84	22.25±3.48	0.0117
Habit			
Smoking	299(83.99)	1346(86.39)	0.2390
Betel nut	247(69.38)	1137(72.98)	0.1713
alcohol	262(73.60)	1194(76.64)	0.2250
Comorbidities			
Myocardial infarct	21(5.90)	94(6.03)	0.9232
Congestive heart failure	40(11.24)	118(7.57)	0.0235
Peripheral vascular disease	15(4.21)	57(3.66)	0.6195
Cerebrovascular disease	74(20.79)	272(17.46)	0.1410
Dementia	17(4.78)	44(2.82)	0.0586
Chronic lung disease	251(70.51)	883(56.68)	<.0001
Connective tissue disease	10(2.81)	24(1.54)	0.1021
Ulcer	171(48.03)	649(41.66)	0.0282
Chronic liver disease	68(19.1)	261(16.75)	0.2892
Diabetes	120(33.71)	354(22.72)	<.0001
Diabetes with end organ damage	41(11.52)	100(6.42)	0.0009

Moderate or severe kidney disease	24(6.74)	104(6.68)	0.9639
Death	197(55.34)	845(54.24)	0.7067

Abbreviations: BMI: body mass index; CYP: cytochrome P450; EGFR: epidermal growth factor receptor; TKI: tyrosine kinase inhibitor; P-gp: P-glycoprotein; PPI: proton pump inhibitor

Supplementary Table S2. Prediction for occurrence of death (unmatched comparison)

	Crude		Adjusted	
	HRs	p-value	HRs	p-value
rifamycin vs. non-rifamycin	1.12(0.81-1.54)	0.4886	1.33(0.93-1.90)	0.1238
Age	1.01(0.99-1.02)	0.5326	1.01(0.99-1.03)	0.4180
Male vs. Female	1.48(0.99-2.23)	0.0562	1.36(0.87-2.12)	0.1737
Medications				
CYP inducer	2.07(1.27-3.37)	0.0033	2.73(1.54-4.85)	0.0006
CYP inhibitor	1.42(0.98-2.07)	0.0661	1.40(0.94-2.09)	0.0995
P-gp inducer	1.82(1.03-3.21)	0.0400	1.17(0.61-2.25)	0.6345
P-gp inhibitor	1.18(0.72-1.93)	0.5166	1.42(0.79-2.55)	0.2433
PPI	0.97(0.69-1.37)	0.8504	0.85(0.57-1.26)	0.4079
EGFR TKI	1.90(1.29-2.80)	0.0013	1.79(1.18-2.73)	0.0065
Lung cancer stages				
Stage 1&2	REF.		REF.	
Stage 3	5.54(1.97-15.6)	0.0011	4.65(1.60-13.5)	0.0048
Stage 4	3.85(1.42-10.5)	0.0081	3.13(1.10-8.88)	0.0320
Unknow	1.32(0.24-7.21)	0.7478	1.35(0.24-7.64)	0.7364
Comorbidities				
Myocardial infarct	0.74(0.36-1.52)	0.4146	0.85(0.38-1.89)	0.6893
Congestive heart failure	0.98(0.59-1.65)	0.9494	1.19(0.64-2.22)	0.5862
Peripheral vascular disease	1.31(0.61-2.80)	0.4830	1.16(0.46-2.90)	0.7544
Cerebrovascular disease	0.67(0.44-1.03)	0.0673	0.58(0.33-1.02)	0.0588
Dementia	0.69(0.25-1.86)	0.4596	0.91(0.29-2.87)	0.8743
Chronic lung disease	1.27(0.90-1.80)	0.1681	1.41(0.97-2.07)	0.0734
Connective tissue disease	1.13(0.46-2.76)	0.7850	1.49(0.58-3.81)	0.4045
Ulcer	0.96(0.70-1.32)	0.7840	0.87(0.62-1.23)	0.4399
Chronic liver disease	0.76(0.48-1.19)	0.2307	0.80(0.48-1.31)	0.3701
Diabetes	0.89(0.62-1.29)	0.5458	0.99(0.63-1.53)	0.9496
Diabetes with end organ damage	0.48(0.22-1.02)	0.0550	0.42(0.18-1.01)	0.0525
Hemiplegia	1.39(0.44-4.35)	0.5743	1.60(0.44-5.79)	0.4726
Moderate or severe kidney disease	1.52(0.82-2.81)	0.1825	2.17(1.07-4.40)	0.0328

Abbreviations: BMI: body mass index; CYP: cytochrome P450; EGFR: epidermal growth factor receptor; TKI: tyrosine kinase inhibitor; P-gp: P-glycoprotein; PPI: proton pump inhibitor

Supplementary Table S3. Baseline Characteristics of the liver injury population

	Non-rifamycin N=62(26.16%)	rifamycin N=175(73.84%)	p-value
Age	69.85±11.33	68.64±11.65	0.4784
Age Group			0.3977
20-59	11(17.74)	43(24.57)	
60-75	23(37.10)	68(38.86)	
>75	28(45.16)	64(36.57)	
Gender			0.4053
Female	13(20.97)	46(26.29)	
Male	49(79.03)	129(73.71)	
Medications			
CYP inducer	7(11.29)	12(6.86)	0.2693
CYP inhibitor	17(27.42)	22(12.57)	0.0067
P-gp inducer	4(6.45)	8(4.57)	0.5618
P-gpinhibitor	7(11.29)	13(7.43)	0.3472
PPI	23(37.1)	63(36)	0.8773
EGFR TKI	4(6.45)	24(13.71)	0.1279
Lung cancer stages			0.1847
Stage 1 &2	13(20.97)	21(12.00)	
Stage 3	15(24.19)	33(18.86)	
Stage 4	30(48.39)	103(58.86)	
Unknow	4(6.45)	18(10.29)	
Histology			0.9010
Adenocarcinoma	26(42.62)	85(49.13)	
Squamous cell carcinoma	19(31.15)	46(26.59)	
Small cell carcinoma	3(4.92)	9(5.20)	
Unclassified malignancy	8(13.11)	18(10.4)	
Other histological types	5(8.20)	15(8.67)	
BMI	21.53±3.26	23.58±4.21	0.0299
Habit			
Smoking	50(80.65)	139(79.43)	0.8377
Betel nut	40(64.52)	114(65.14)	0.9292
alcohol	39(62.9)	125(71.43)	0.2115
Comorbidities			
Myocardial infarct			
Congestive heart failure	6(9.68)	18(10.29)	0.8915
Peripheral vascular disease	3(4.84)	7(4.00)	0.7777
Cerebrovascular disease	12(19.35)	44(25.14)	0.3566
Dementia	3(4.84)	8(4.57)	0.9315
Chronic lung disease	42(67.74)	115(65.71)	0.7717
Connective tissue disease	4(6.45)	5(2.86)	0.2032
Ulcer	40(64.52)	106(60.57)	0.5831
Chronic liver disease	24(38.71)	75(42.86)	0.5694
Diabetes	17(27.42)	57(32.57)	0.4519
Diabetes with end organ damage	5(8.06)	15(8.57)	0.9018

Moderate or severe kidney disease	5(8.06)	17(9.71)	0.7005
Death	37(59.68)	98(56.00)	0.6153

Abbreviations: BMI: body mass index; CYP: cytochrome P450; EGFR: epidermal growth factor receptor; TKI: tyrosine kinase inhibitor; P-gp: P-glycoprotein; PPI: proton pump inhibitor

Supplementary Table S4. Prediction for occurrence of death in liver injury population

	Crude		Adjusted	
	HRs	p-value	HRs	p-value
rifamycin vs. Non-rifamycin	0.94(0.64-1.37)	0.7363	0.91(0.6-1.39)	0.6597
Age	1(0.99-1.02)	0.5651	1(0.98-1.02)	0.9779
Male vs. Female	1.09(0.74-1.61)	0.654	1.04(0.68-1.6)	0.8515
Medications				
CYP inducer	1.01(0.56-1.83)	0.969	0.94(0.46-1.92)	0.8756
CYP inhibitor	1.03(0.66-1.61)	0.9085	0.99(0.59-1.66)	0.9834
P-gp inducer	1.43(0.7-2.92)	0.3263	2.39(1.02-5.59)	0.0438
P-gp inhibitor	2.32(1.38-3.87)	0.0014	2.86(1.58-5.15)	0.0005
PPI	1.16(0.82-1.64)	0.3941	1.33(0.88-2.01)	0.1804
EGFR TKI	1.31(0.81-2.1)	0.2696	1.14(0.66-1.96)	0.6483
Lung cancer stages				
Stage 1&2	REF.		REF.	
Stage 3	1.5(0.79-2.86)	0.2124	1.79(0.91-3.54)	0.0923
Stage 4	1.84(1.05-3.25)	0.0346	2.32(1.22-4.38)	0.0099
Unknow	1.19(0.54-2.62)	0.6657	1.5(0.66-3.43)	0.3367
Comorbidities				
Myocardial infarct	1.3(0.72-2.35)	0.3904	1.31(0.65-2.63)	0.4517
Congestive heart failure	1.05(0.62-1.77)	0.8572	0.92(0.51-1.68)	0.7952
Peripheral vascular disease	1.39(0.65-2.97)	0.3986	1.25(0.5-3.15)	0.6345
Cerebrovascular disease	1.12(0.76-1.65)	0.569	1.1(0.67-1.83)	0.7047
Dementia	2.15(1.09-4.24)	0.0267	1.49(0.64-3.45)	0.357
Chronic lung disease	1.39(0.95-2.02)	0.0872	1.54(1.01-2.33)	0.0429
Connective tissue disease	0.96(0.39-2.35)	0.9308	1.37(0.46-4.12)	0.5705
Ulcer	0.81(0.58-1.14)	0.2326	0.71(0.48-1.06)	0.0927
Chronic liver disease	0.77(0.54-1.09)	0.1446	0.81(0.54-1.21)	0.3079
Diabetes	0.93(0.65-1.35)	0.7089	0.95(0.6-1.52)	0.8424
Diabetes with end organ damage	0.98(0.53-1.82)	0.9482	1.14(0.54-2.42)	0.7337
Hemiplegia	0.76(0.19-3.06)	0.696	0.55(0.12-2.53)	0.4394
Moderate or severe kidney disease	0.67(0.34-1.32)	0.2445	0.49(0.22-1.08)	0.0757
Moderate or severe liver disease	1.33(0.42-4.18)	0.6266	1.16(0.35-3.86)	0.8141

Abbreviations: BMI: body mass index; CYP: cytochrome P450; EGFR: epidermal growth factor receptor; TKI: tyrosine kinase inhibitor; P-gp: P-glycoprotein; PPI: proton pump inhibitor
